# Nourishing Holistic Well-Being: The Role of Family Dynamics and Family Cooking

**DOI:** 10.3390/healthcare13040414

**Published:** 2025-02-14

**Authors:** Yen-Cheng Chen, Ching-Sung Lee, Ming-Chen Chiang, Pei-Ling Tsui, Bo-Kai Lan, Yi-Jie Chen

**Affiliations:** 1 Department of Applied Science of Living, Chinese Culture University, Taipei 11114, Taiwan; cyc4@ulive.pccu.edu.tw (Y.-C.C.); lanbaikai@gmail.com (B.-K.L.); b1708100@ulive.pccu.edu.tw (Y.-J.C.); 2 Department of Restaurant, Hotel and Institutional Management, Fu Jen Catholic University, New Taipei City 242062, Taiwan; 3 Ph. D. Program in Nutrition and Food Science, College of Human Ecology, Fu Jen Catholic University, New Taipei City 242062, Taiwan; 4 Department of Hospitality Management, National Taitung Junior College, Taitung 95045, Taiwan; 5 Graduate Institute of Technological and Vocational Education, National Taipei University of Technology, Taipei 10608, Taiwan

**Keywords:** family function, psychological flourishing, family well-being, shared cooking and dining practices, family-centered interventions

## Abstract

**Background:** Family interactions play a pivotal role in shaping well-being across physical, psychological, and social domains. While substantial research has focused on the nutritional and physical health benefits of family meals, the impact of shared cooking and dining practices on psychological flourishing and overall family well-being remains underexplored. **Objectives:** This study examines the relationships among family function, shared culinary practices, psychological flourishing, and family well-being, with particular emphasis on the mediating and moderating roles of psychological flourishing. **Methods:** A cross-sectional survey was conducted involving 461 participants from Chinese families with diverse family structures in Taipei, Taiwan. Data were analyzed using multiple regression analysis, as well as mediation and moderation analyses, to assess the interrelationships between family function, shared cooking and dining practices, and family well-being. **Results:** The findings indicate that family function significantly enhances psychological flourishing, which, in turn, mediates the association between family function and family well-being. Furthermore, shared cooking and dining practices were found to positively influence family well-being, underscoring their critical role in strengthening emotional bonds, fostering communication, and improving family cohesion. **Conclusions:** This study contributes to family nursing science and positive psychology by highlighting shared cooking and dining practices as effective interventions for enhancing family well-being. By incorporating the concept of psychological flourishing, this research expands our understanding of how family dynamics and shared culinary activities contribute to emotional resilience and life satisfaction. **Implications:** Given the cross-sectional nature of the study, future research should employ longitudinal designs to explore causal relationships and the long-term effects of family interactions on well-being. Practical recommendations include the promotion of family-centered nutrition education programs and the development of public policies that encourage shared family meals as a strategy to enhance both emotional health and physical well-being.

## 1. Introduction

Family interactions are a cornerstone of well-being, influencing physical, mental, and social dimensions across the lifespan. Positive family dynamics foster emotional resilience, life satisfaction, and social harmony, enabling individuals to develop behavioral norms, emotional regulation skills, and collaborative problem-solving abilities [1]. These interactions contribute to holistic health by providing a supportive environment for personal growth and collective stability. Empirical evidence consistently demonstrates that robust family relationships are strongly associated with improved mental health outcomes, including reduced anxiety and depression, and enhanced coping mechanisms during adversity [2,3].

Structured family activities, such as shared leisure pursuits, collaborative tasks, and participatory meal preparation, play a pivotal role in promoting family cohesion, enhancing communication, and strengthening emotional bonds [4,5,6]. Among these, family cooking and dining practices have garnered significant attention due to their profound impact on familial relationships. Shared meal preparation and dining offer a structured yet adaptable context for fostering interpersonal connections while supporting both emotional and physical health. Studies reveal that regular family meals improve dietary quality, mitigate unhealthy behaviors, and reinforce familial ties [7,8].

However, contemporary lifestyles—marked by time constraints, reliance on convenience foods, and conflicting schedules—have contributed to a decline in the frequency of family meals. Research indicates that the frequency of family meals has steadily decreased by more than 20–30% over recent decades, particularly in Western and industrialized countries [7]. This decline in shared family meals has raised concerns about its implications for family cohesion and well-being, with studies linking a reduced family meal frequency to negative health outcomes such as poor dietary habits, weight gain, and emotional distress [8,9]. Meals consumed outside the home often lack the nutritional balance necessary for optimal health, heightening the risk of obesity, chronic diseases, and mental health challenges. These challenges underscore the urgent need to re-examine family practices as a mechanism for promoting sustainable health outcomes and fostering psychological well-being.

From a psychological perspective, family cooking and dining practices extend beyond meeting nutritional needs to address the broader dimensions of well-being through the lens of flourishing. Flourishing, a concept rooted in positive psychology, encompasses positive emotions, meaningful relationships, and personal growth [10]. Collaborative culinary activities foster mutual support, teamwork, and a sense of accomplishment, which collectively enhance emotional resilience and life satisfaction [11,12,13]. Within the familial context, shared meals provide a unique platform for strengthening relational bonds and creating a nurturing environment conducive to holistic health. Moreover, these practices contribute to psychological flourishing by fostering shared emotional experiences, providing a sense of meaning, and reinforcing family members’ mutual sense of purpose and support [14].

Nutritionally, family meals are instrumental in fostering healthier eating behaviors, promoting balanced nutrition, and mitigating the risks associated with poor dietary habits. Regular family meals have been consistently associated with an improved dietary quality, better weight management, and a lower incidence of chronic conditions such as diabetes and cardiovascular diseases [1,7,15]. Additionally, family meals play a critical role in preserving cultural traditions, transmitting intergenerational knowledge, and fostering social cohesion, which contributes to a shared sense of identity and belonging.

Despite the established benefits of family meals, there remains a significant gap in the literature regarding their role as a moderator in the relationship between family function and well-being. While much of the existing research has focused on the nutritional outcomes of family meals or their impact on physical health, fewer studies have examined the psychosocial dimensions, specifically how shared cooking and dining practices influence family dynamics, psychological flourishing, and overall family well-being. This gap in the literature highlights the necessity of a comprehensive investigation into the interplay between family dynamics, shared cooking practices, and psychological well-being. Moreover, most studies have focused on isolated health outcomes, overlooking the moderating role of these practices in enhancing family cohesion and emotional resilience.

By adopting a systems-oriented perspective, this study aims to address these gaps by examining how family function, shared cooking and dining practices, and psychological flourishing influence family well-being. Systems theory posits that family members are interconnected, and changes in one member’s behaviors or emotions can affect the family as a whole. This perspective is particularly relevant when examining how shared culinary practices can influence family dynamics and well-being. Additionally, positive psychology provides a framework for understanding how cultivating positive emotions, meaningful relationships, and personal growth within family interactions contributes to overall flourishing.

This study’s findings are anticipated to contribute to the advancement of family nursing science and positive psychology, offering evidence-based insights for interventions that promote family cohesion, self-actualization, and sustainable health outcomes. By focusing on the psychosocial dimensions of family meals, it emphasizes the importance of shared culinary practices not only in improving physical health but also in enhancing emotional resilience and holistic family well-being.

### Research Objectives

This study explores the impact of family cooking and dining practices on holistic family well-being, focusing on their moderating role in the relationship between family dynamics and psychological flourishing. By addressing gaps in understanding the interplay between shared culinary activities and well-being, the study aims to:

1. Examine how family dynamics and shared culinary practices influence dietary quality, emotional resilience, and social cohesion.
2. Investigate the relationships among family dynamics, cooking and dining practices, and family well-being.
3. Assess the contributions of shared culinary activities to balanced nutrition, psychological flourishing, and relational satisfaction.
4. Evaluate the moderating effect of cooking and dining practices on the relationship between family dynamics and family well-being.

The findings aim to provide empirical evidence for the role of shared culinary practices in fostering cohesive and resilient family systems, informing family-centered interventions that promote holistic well-being through sustainable practices.

## 2. Literature Review and Hypothesis Development

### 2.1. Family Dynamics

Family dynamics encompass the patterns of interaction, relationships, and roles within a family system that shape members’ behaviors, emotions, and overall well-being. A well-functioning family fosters emotional support, stability, and a nurturing environment, which are crucial for promoting both individual and collective health [16]. According to family systems theory, family members are interdependent, meaning that changes in one member’s behavior or well-being can influence the entire family unit. This interconnectivity highlights how family functionality directly affects physical, mental, and social health outcomes [17].

Research highlights the critical role of cohesion and adaptability in fostering health-promoting behaviors. Cohesion, defined as the emotional bonding among family members, and adaptability, the ability to manage changes and challenges, are positively associated with dietary quality and mental resilience [18]. Families with high levels of cohesion and adaptability are more likely to engage in shared meal practices, which contribute to improved nutritional choices, enhanced emotional well-being, and stronger social bonds [19]. These mechanisms operate through pathways such as stress reduction, emotional regulation, and improved conflict resolution, which in turn facilitate healthier eating habits and psychological stability.

However, modern challenges such as fragmented schedules, pervasive technology use, and an increased reliance on convenience foods have strained family dynamics, limiting opportunities for meaningful interactions and shared health-promoting behaviors [9,20]. These disruptions have been linked to decreased family cohesion, poor dietary patterns, and heightened psychological distress, particularly among families experiencing time constraints or economic hardship [21]. Cultural norms and socio-economic conditions further influence family dynamics, with low-income families facing greater barriers to home-cooked meals due to limited financial resources, time, and access to fresh ingredients. In contrast, cultures that emphasize communal eating and intergenerational meal preparation may sustain stronger family bonds despite external challenges.

Despite these barriers, evidence suggests that strong family dynamics can mitigate adverse health outcomes by promoting balanced nutrition and mindful dietary practices. Families that prioritize open communication, emotional support, and shared responsibilities are more likely to prepare home-cooked meals, which have been associated with an improved dietary quality, a reduced risk of chronic diseases, and enhanced social connections [22]. Furthermore, family cohesion serves as a protective factor against the negative impacts of dietary imbalances and stress, supporting both physical and mental health [23].

Understanding the mechanisms through which family dynamics influence nutrition and holistic health is essential for developing effective interventions. By recognizing how emotional bonding, adaptability, and communication shape dietary behaviors, public health initiatives can better support families in adopting sustainable health-promoting practices.

Based on the literature, the following hypothesis is proposed:

**H1.**  *Strong family dynamics positively influence dietary behaviors and overall family well-being, with higher levels of cohesion and adaptability associated with improved physical, mental, and social health outcomes.*

### 2.2. Family Cooking and Dining Practices

Family cooking and dining practices encompass the preparation and sharing of meals within a household—activities that hold both practical and symbolic significance. These practices serve as vital platforms for fostering family cohesion, encouraging healthy dietary behaviors, and promoting overall well-being. Beyond their nutritional role, shared meals are deeply embedded in cultural traditions, functioning as a medium for transmitting values, identity, and intergenerational knowledge. Practically, these activities promote teamwork, shared responsibility, and mutual trust, strengthening relational bonds and facilitating open communication within the family [5]. Research consistently demonstrates that families who dine together more frequently report higher levels of emotional closeness, life satisfaction, and a greater sense of accomplishment [6,24,25,26].

Family meals are strongly associated with improved dietary quality and health outcomes. Regularly shared meals promote a higher consumption of fruits, vegetables, and whole grains, while reducing the intake of processed foods and sugary beverages [7]. Additionally, these practices act as protective factors against obesity and related health risks, supporting healthier eating habits through modeling behaviors, routine establishment, and structured meal environments [27]. Psychologically, shared meals contribute to well-being by reducing stress, enhancing emotional resilience, and fostering relational harmony [15]. The mechanisms underlying these effects involve stress alleviation, emotional support, and conflict resolution, which collectively improve psychological flourishing and family functioning.

Despite their benefits, family cooking and dining practices face significant challenges in modern contexts. Time constraints, an increased reliance on convenience foods, and individualized eating patterns have contributed to a decline in shared meals, disrupting traditional family routines and limiting opportunities for face-to-face interactions [9,28]. Cultural and socio-economic variations further shape these patterns; for example, low-income families may struggle to maintain home-cooked meals due to financial limitations, while cultures that emphasize communal eating and intergenerational cooking may be more resilient to these shifts. The COVID-19 pandemic further underscored the dual nature of shared meals, as they functioned both as a coping mechanism for family stress and, in some cases, as an additional source of strain due to economic hardship or increased caregiving burdens [29].

Cooking as a collaborative activity enhances family functioning by promoting teamwork, creativity, and problem-solving. It provides a structured yet informal setting for interaction, strengthening communication and emotional closeness among family members [24]. Intergenerational cooking reinforces familial relationships, allowing older family members to pass down cultural traditions, share knowledge, and foster a sense of purpose among younger generations [26,30]. Additionally, the benefits of shared cooking and dining practices may vary across family structures. For instance, single-parent households may experience greater difficulty maintaining shared meal routines due to work constraints, while multi-generational families may leverage these practices to strengthen caregiving roles and cultural continuity.

Based on the evidence, family cooking and dining practices are hypothesized to positively influence family well-being by enhancing family cohesion, promoting healthy behaviors, and fostering psychological flourishing.

**H2.**  *Family cooking and dining practices positively influence family well-being, with higher engagement associated with improved psychological flourishing.*

### 2.3. Family Cooking and Dining Practices

Family well-being is a multidimensional construct encompassing the physical, mental, and social health of all family members. It reflects not only individual happiness and resilience but also the collective functionality and harmony of the family unit [16]. Key indicators of family well-being—such as life satisfaction, emotional stability, and relational quality—are shaped by the frequency and quality of family interactions, particularly shared activities [29]. In contemporary family research, shared routines, such as cooking and dining together, are increasingly recognized as foundational elements that promote emotional closeness and social integration.

Psychological flourishing, a central concept in positive psychology, represents an optimal state of well-being characterized by positive emotions, meaningful relationships, and personal growth. Within family contexts, flourishing emerges when members experience emotional support, mutual understanding, and a shared sense of purpose. Such families exhibit stronger communication, adaptability, and conflict resolution skills, all of which contribute to relational stability and overall well-being [11]. Families that prioritize cohesion, open communication, and adaptability create supportive environments that enhance emotional resilience, particularly during times of stress. These environments increase life satisfaction and reduce psychological distress [29].

Shared cooking and dining practices further enhance family well-being by fostering collaboration, emotional connection, and shared achievement. Empirical studies indicate that these activities significantly improve life satisfaction, emotional resilience, and interpersonal relationships across diverse family structures and cultural contexts [19,31,32]. For example, frequent family meals are associated with reduced depressive symptoms and greater overall happiness, reinforcing their role in fostering psychological well-being [22,33]. Additionally, shared cooking and dining practices moderate the relationship between family dynamics and well-being by providing structured opportunities for communication and cooperation. These practices are particularly effective in strengthening relational bonds in families with moderate cohesion, suggesting that collaborative routines help sustain family well-being even in less tightly connected family units [30,31].

Based on the literature, the following hypothesis is proposed:

**H3.**  *Family cooking and dining practices serve as a moderating factor in the relationship between family dynamics and family well-being, amplifying the positive effects of cohesive family interactions on psychological flourishing.*

### 2.4. The Mediating Role of Psychological Flourishing in Family Interactions

Psychological flourishing, defined as an optimal state of well-being, encompasses positive emotions, meaningful relationships, and personal growth [10]. Within family contexts, flourishing functions as both an outcome of positive interactions and a mediating mechanism that connects relational benefits to broader well-being outcomes [11]. Families characterized by open communication, emotional support, and adaptability create environments conducive to flourishing, where positive interactions translate into emotional stability, relational satisfaction, and life satisfaction [16,29]. For example, individuals from supportive family environments consistently report higher levels of flourishing, which in turn enhances their psychological health and overall well-being [19].

Family cooking and dining practices further amplify the mediating role of flourishing in promoting family well-being. These shared activities foster engagement, collaboration, and a sense of achievement, strengthening relational bonds and promoting mindfulness—an essential component of flourishing [22,26]. Research demonstrates that families engaging in frequent meal preparation and dining together experience higher levels of life satisfaction, emotional resilience, and social cohesion, illustrating how collaborative meal routines reinforce psychological well-being [9,30]. Flourishing mediates these effects by bridging the relationship between collaborative family practices and well-being, elucidating the mechanisms through which shared activities contribute to happiness and functionality [34].

Based on the theoretical and empirical evidence, the following hypotheses are proposed:

**H4.**  *Psychological flourishing mediates the relationship between family dynamics and family well-being, such that stronger family dynamics lead to greater flourishing, which in turn enhances family well-being.*

**H5.**  *Psychological flourishing mediates the relationship between family cooking and dining practices and family well-being, such that higher engagement in these practices promotes flourishing, which in turn enhances family well-being.*

## 3. Materials and Methods

### 3.1. Ethical Statement

This study was approved by the National Taiwan University Research Ethics Committee. Prior to data collection, all participants provided written informed consent to ensure voluntary participation. The participants were informed of their rights to withdraw from the study at any point without penalties and assured of the confidentiality and anonymity of their responses.

### 3.2. Research Design and Study Subjects

This study employed a cross-sectional survey, in which participants were asked to complete a structured questionnaire assessing family dynamics, family cooking and dining practices, psychological flourishing, and family well-being. The target population consisted of residents from Taipei City, Taiwan, who met specific inclusion criteria. To ensure data independence, only one participant per household was included in the study. Participants were required to engage in family cooking and dining activities at least twice per week, share meals with a minimum of two co-residing family members, and actively participate in at least one cooking-related activity, such as menu planning, ingredient preparation, cooking, table setting, or post-meal cleaning. These criteria ensured that participants were meaningfully involved in family-oriented culinary practices, thereby aligning with the study’s objectives and ensuring relevant, high-quality data for analysis.

### 3.3. Sampling Methodology

Data collection utilized a combination of purposive and snowball sampling methods to recruit participants who met the inclusion criteria. Recruitment announcements were disseminated through community centers, social networks, and online platforms to ensure a diverse participant pool. This multi-faceted recruitment strategy facilitated the identification of participants who were actively engaged in family culinary practices, while also ensuring demographic and socio-cultural diversity within Chinese families in Taipei.

### 3.4. Sample Size and Demographics

A total of 461 valid responses were included in the final analysis. The sample predominantly comprised individuals aged 41–50 years (52.93%), with over half of the participants (58.35%) belonging to nuclear families with children and engaging in family cooking and dining activities at least twice weekly. Additionally, the sample included a range of Chinese family structures and socio-economic backgrounds, ensuring a representative dataset for examining the relationships between family dynamics, psychological flourishing, and family well-being. These characteristics provide a strong foundation for analyzing the impact of shared culinary practices within diverse Chinese family contexts.

### 3.5. Family Function Scale

The Family Function Scale was developed based on Olson’s Circumplex Model of Marital and Family Systems, integrating the dimensions of family cohesion and adaptability. Adapted from the Family Cohesion and Adaptability Evaluation Scale (FACES), the instrument was modified to assess two dimensions: intimacy and harmony and flexibility and adaptability. The scale consisted of 16 items rated on a 5-point Likert scale, ranging from 1 = Strongly Disagree to 5 = Strongly Agree. It demonstrated excellent reliability, with a Cronbach’s α of 0.93. Specifically, the intimacy and harmony dimension exhibited a Cronbach’s α of 0.935, while the flexibility and adaptability dimension showed a Cronbach’s α of 0.812, indicating high internal consistency across items.

Exploratory factor analysis (EFA) confirmed the structural validity of the scale. The Kaiser–Meyer–Olkin (KMO) measure of sampling adequacy was 0.895, and Bartlett’s test of sphericity was significant (χ^2^ = 1135.451, df = 120, *p* < 0.001), validating the dataset’s suitability for factor analysis. Two distinct factors emerged, explaining 60.711% of the total variance. The flexibility and adaptability dimension accounted for 39.305% of the variance, focusing on problem-solving and emotional regulation, while the intimacy and harmony dimension accounted for 21.406% of the variance, emphasizing emotional closeness and shared family activities.

These findings, summarized in Table 1, highlight the robust psychometric properties of the scale. The high reliability and structural validity validate its effectiveness in assessing family dynamics, particularly in understanding how family interactions impact psychological and relational well-being. The strong factor loadings further support its applicability in examining the role of family cohesion and adaptability in promoting family well-being, reinforcing its relevance to the study’s objectives.

### 3.6. Family Cooking and Dining Practices Scale

The Family Cooking and Dining Practices Scale was developed based on prior research on family rituals and shared culinary activities, including the works of Fulkerson et al. [7] and Neumark-Sztainer et al. [9]. These studies highlighted the role of shared meals and collaborative cooking in fostering family cohesion, improving communication, and promoting emotional well-being. Drawing from this foundation, the current scale was designed to measure two key dimensions: interaction quality and participation frequency; these captured both relational engagement and habitual involvement in family meal practices. The scale consisted of 10 items rated on a 5-point Likert scale, ranging from 1 = Strongly Disagree to 5 = Strongly Agree. It demonstrated excellent reliability, with a Cronbach’s α of 0.87.

Exploratory factor analysis (EFA) was conducted to validate the structural integrity of the scale. The Kaiser–Meyer–Olkin (KMO) measure of sampling adequacy was 0.911, and Bartlett’s test of sphericity was significant (χ^2^ = 1387.623, df = 136, *p* < 0.001), confirming that the dataset was appropriate for factor analysis. Two factors were extracted, cumulatively explaining 65.382% of the total variance, supporting the scale’s validity for assessing culinary practices within the family context.

The interaction quality dimension captured the relational and emotional benefits of shared cooking and dining. For example, cooking together was linked to improved family communication and teamwork, as reflected in high factor loadings such as 0.874 for “Cooking together strengthens our family bond”. Meanwhile, the participation frequency dimension emphasized the habitual nature of these practices, including regular meal planning and shared cooking responsibilities, with a loading of 0.712 for “Our family eats together at least three times a week”. These findings align with existing theories on family rituals, reinforcing the scale’s reliability in evaluating the impact of shared culinary experiences on family well-being.

The detailed factor loadings and associated statistics are presented in Table 2, highlighting the scale’s robust psychometric properties. By capturing both relational and habitual dimensions, this scale provides a comprehensive measure of the role of family cooking and dining practices in fostering relational harmony, strengthening communication, and enhancing emotional engagement.

### 3.7. Psychological Flourishing Scale

The Psychological Flourishing Scale was adapted from Diener et al. [35], emphasizing the dimensions of positive emotions, engagement, meaning, and relational well-being. This scale was designed to measure individuals’ perceptions of purpose, personal growth, and emotional connections within their family context, aligning with the study’s focus on family well-being. The scale consisted of 8 items rated on a 5-point Likert scale, ranging from 1 = Strongly Disagree to 5 = Strongly Agree. It demonstrated excellent reliability, with a Cronbach’s α of 0.94.

Exploratory factor analysis (EFA) was conducted to validate the construct validity of the scale. The Kaiser–Meyer–Olkin (KMO) measure of sampling adequacy was 0.895, and Bartlett’s test of sphericity yielded significant results (χ^2^ = 1042.263, df = 28, *p* < 0.001), confirming the dataset’s suitability for factor analysis. A single factor was extracted, explaining 63.178% of the total variance, indicating that the scale effectively measures a unidimensional construct of psychological flourishing.

The factor loadings ranged from 0.742 to 0.856, with items such as “I feel that I am a part of something greater than myself” showing the highest loading, at 0.856. This finding underscores the importance of relational and emotional well-being within the family context, aligning with the theoretical framework of flourishing. Other high-loading items, such as “My relationships contribute to my personal growth”, reinforce the connection between personal development and family relational dynamics.

The detailed factor loadings and statistical results are presented in Table 3, further validating the scale’s robust psychometric properties. By capturing a unified construct of flourishing, the scale provides a comprehensive assessment of how shared experiences and positive interactions within families enhance psychological well-being.

### 3.8. Family Well-Being Scale

The Family Well-Being Scale was developed based on theoretical frameworks and empirical studies addressing family satisfaction, relational harmony, and overall happiness. Drawing on the works of Walsh [16] and Olson et al. [36], the scale integrates the concepts of family cohesion and emotional well-being, providing a comprehensive measure of holistic family health. This instrument was adapted to assess the extent to which family members experience harmony, support, and happiness within their family context. The scale consisted of 10 items rated on a 5-point Likert scale, ranging from 1 = Strongly Disagree to 5 = Strongly Agree. It demonstrated excellent reliability, with a Cronbach’s α of 0.94.

Exploratory factor analysis (EFA) was conducted to evaluate the construct validity of the scale. The Kaiser–Meyer–Olkin (KMO) measure of sampling adequacy was 0.902, and Bartlett’s test of sphericity was significant (χ^2^ = 975.412, df = 66, *p* < 0.001), confirming the dataset’s suitability for factor analysis. A single factor was extracted, explaining 62.385% of the total variance, indicating that the scale effectively measures a unidimensional construct of family well-being.

The factor loadings ranged from 0.689 to 0.841, with items such as “I feel happy and satisfied with my family life” exhibiting the highest loading at 0.841. This finding underscores the central role of emotional satisfaction in family well-being. Other high-loading items, such as “My family has a harmonious relationship”, reinforce the importance of relational harmony in contributing to overall happiness within the family.

The detailed factor loadings and statistical results are presented in Table 4, underscoring the scale’s robust psychometric properties. By providing a comprehensive measure of family happiness and harmony, the scale offers valuable insights into relational dynamics and emotional satisfaction within families, reinforcing its relevance to the study’s exploration of family interactions and psychological flourishing.

### 3.9. Data Analysis

Data were analyzed using SPSS version 26 to examine the relationships among family dynamics, family cooking and dining practices, psychological flourishing, and family well-being. Descriptive statistics summarized the demographic characteristics and variable distributions. Reliability and validity were assessed through Cronbach’s α and exploratory factor analysis (EFA) to confirm internal consistency and construct validity. Pearson’s correlation analysis explored associations among the primary constructs.

Multiple regression analyses tested the predictive effects of family dynamics and family cooking and dining practices on psychological flourishing and family well-being. Mediation and moderation analyses were conducted using the PROCESS macro in SPSS to examine the mediating and moderating roles of psychological flourishing. Additionally, one-way ANOVA identified differences in family well-being and psychological flourishing across demographic groups (e.g., age, cooking frequency). Data screening procedures addressed missing values, normality, and multicollinearity, ensuring the robustness of the findings.

## 4. Results

### 4.1. Demographic Data of the Subjects

A total of 461 valid responses were included in the final analysis. The demographic characteristics of the participants are summarized in Table 5. The majority of the sample comprised individuals aged 41–50 years (52.93%, *n* = 323), followed by those aged 31–40 years (27.46%, *n* = 138). The gender distribution was predominantly female (68.12%, *n* = 314), with males accounting for 31.88% (*n* = 147).

In terms of family structure, 58.35% (*n* = 269) of the participants belonged to nuclear families, while 41.65% (*n* = 192) were part of extended families. Regarding educational attainment, 63.57% (*n* = 293) held a university degree or higher, whereas 36.43% (*n* = 168) had an education level below university.

The frequency of participation in family cooking and dining activities varied among the participants. A significant proportion (75%, *n* = 346) reported engaging in these activities three or more times per week, while 25% (*n* = 115) participated less frequently. These demographic characteristics provide context for understanding family dynamics, psychological flourishing, and family well-being in this study.

### 4.2. The Effect of Family Function on Psychological Flourishing

The multiple regression analysis revealed that family function had a significant positive effect on psychological flourishing (β = 0.45, *t* = 8.42, *p* < 0.001). The model explained 32% of the variance (R^2^ = 0.32), indicating that family function plays a critical role in shaping individuals’ psychological flourishing (Table 6). These findings support Hypothesis H1 and highlight the importance of cohesive and adaptable family dynamics in fostering individuals’ sense of purpose, engagement, and emotional well-being. This result aligns with prior research (e.g., [16]), which underscores the role of family support and relational harmony in enhancing psychological health.

### 4.3. The Effect of Family Cooking and Dining Practices on Family Well-Being

The multiple regression analysis showed that family cooking and dining practices had a significant positive impact on family well-being (β = 0.38, *t* = 7.64, *p* < 0.001). The model explained 27% of the variance (R^2^ = 0.27), suggesting that engaging in shared cooking and dining activities plays a key role in promoting family happiness and relational harmony (Table 7). These findings support Hypothesis H2 and highlight the importance of shared culinary activities in fostering emotional bonds, relational satisfaction, and collective well-being within families. This result is consistent with prior studies (e.g., [7,9]), which emphasize the relational and emotional benefits of family meals.

### 4.4. Mediation Analysis: The Mediating Role of Psychological Flourishing

Mediation analysis revealed that family function had a significant direct effect on family well-being (β = 0.34, *t* = 7.12, *p* < 0.001). Additionally, family function significantly predicted psychological flourishing (β = 0.45, *t* = 8.42, *p* < 0.001), and psychological flourishing positively influenced family well-being (β = 0.29, *t* = 6.25, *p* < 0.001). The indirect effect of family function on family well-being through psychological flourishing was significant, as indicated by the 95% confidence interval (CI) [0.12, 0.28], which did not include zero (Table 8). These findings suggest that psychological flourishing partially mediates the relationship between family function and family well-being, supporting Hypothesis H4. This result highlights the critical role of psychological flourishing in linking family dynamics to overall well-being, aligning with prior research on the interplay between relational harmony and individual growth (e.g., [16,35]).

### 4.5. Moderation Analysis: The Moderating Role of Psychological Flourishing

Moderation analysis showed that psychological flourishing significantly moderated the relationship between family function and family well-being (β = 0.15, *t* = 3.45, *p* < 0.001), indicating that the positive association between family function and family well-being was stronger when psychological flourishing was higher (Table 9).

Simple slopes analysis further demonstrated that when psychological flourishing was high (1 SD above the mean), the effect of family function on family well-being was stronger (β = 0.48, *p* < 0.001). In contrast, when psychological flourishing was low (1 SD below the mean), the effect was weaker but still significant (β = 0.28, *p* < 0.01).

These findings suggest that psychological flourishing enhances the positive impact of family function on well-being, reinforcing its role as a protective factor in family systems.

**Figure 1:**
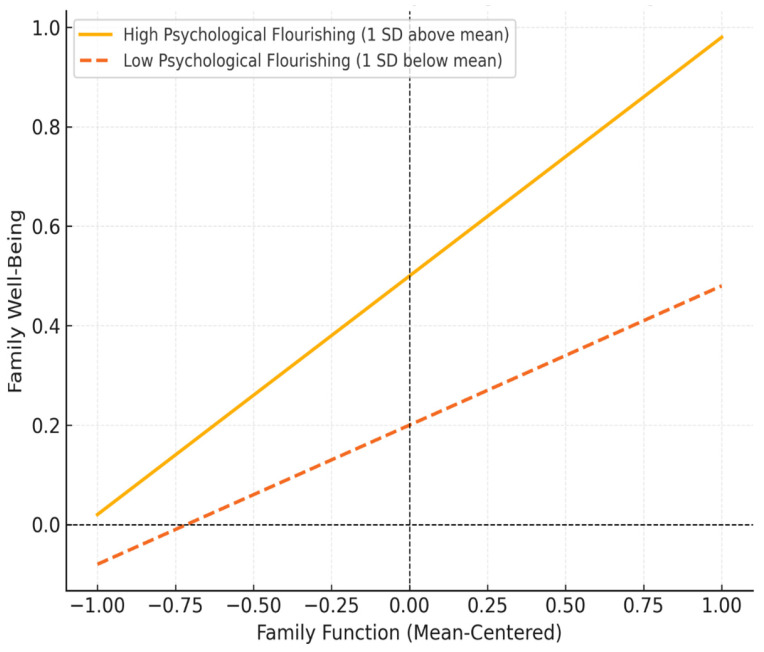
Moderation effect of psychological flourishing.

These findings support Hypothesis H5, demonstrating that psychological flourishing enhances the positive impact of family function on family well-being. Families with high levels of psychological flourishing are better equipped to translate cohesive and adaptive dynamics into greater collective well-being, reinforcing the role of flourishing as a critical buffer in relational contexts. Figure 1 illustrates this moderation effect, showing that the positive association between family function and family well-being is stronger when psychological flourishing is high (1 SD above the mean) compared to when it is low (1 SD below the mean).

## 5. Discussion

This study examined the relationships among family function, family cooking and dining practices, psychological flourishing, and family well-being, emphasizing the family as a holistic system in family nursing science. The findings provide strong empirical support for the proposed hypotheses and offer insights for developing family-centered interventions that enhance family functioning and holistic well-being.

The results indicate that family function has a substantial positive influence on psychological flourishing, supporting Hypothesis H1. This aligns with previous research emphasizing the role of family cohesion and adaptability in fostering psychological resilience and cognitive well-being. Families with effective communication and mutual support provide nurturing environments that enhance both individual and collective well-being [37,38,39].

Family cooking and dining practices were also found to significantly enhance family well-being, corroborating Hypothesis H2. Shared meals create structured opportunities for emotional bonding, meaningful communication, and the reinforcement of health-promoting dietary behaviors. These practices not only improve nutritional quality but also contribute to stress reduction and greater life satisfaction [40,41,42]. However, their impact may vary across different family structures and cultural contexts, suggesting a need for further research on how these practices function in diverse socioeconomic and intergenerational settings.

Psychological flourishing was identified as a mediator in the relationship between family function and family well-being, supporting Hypothesis H4. This suggests that strong family dynamics foster positive psychological states, which, in turn, enhance holistic well-being. The mediating role of psychological flourishing underscores its significance in translating family interactions into tangible health benefits, aligning with prior research on the interplay between relational harmony and individual growth [43].

Furthermore, moderation analysis revealed that psychological flourishing amplifies the positive impact of family function on family well-being, supporting Hypothesis H5. Families with higher psychological flourishing are better positioned to leverage cohesive relational dynamics for greater collective well-being. This interdependence between individual psychological health and family functioning has important implications for family nursing interventions aimed at strengthening psychological resilience and relational harmony.

These findings reinforce the importance of shared family experiences in fostering self-actualization, holistic well-being, and sustainable family health outcomes.

### 5.1. Theoretical Implications

The findings advance theoretical frameworks in family nursing by clarifying the dual role of psychological flourishing as both a mediator and moderator in the relationship between family function and family well-being. While prior research has identified these relationships, this study highlights their systemic nature, emphasizing the interdependence between individual psychological health and family interactions in shaping holistic well-being [44]. These insights strengthen systems-oriented perspectives in family nursing, offering a more comprehensive framework for understanding how family dynamics contribute to both individual and collective health outcomes.

Additionally, integrating family cooking and dining practices into family health discourse enriches existing theoretical models by addressing often-overlooked social and cultural dimensions of well-being. Shared culinary practices provide a dynamic context for fostering interpersonal relationships, enhancing family cohesion, and transmitting shared values, bridging a critical gap in traditional health models. This perspective aligns with emerging paradigms in nutrition science and positive psychology, which emphasize a holistic view of health that incorporates physical, mental, and social dimensions [45]. By positioning shared meals within family nursing theory, this study offers new pathways for conceptualizing interventions that address multiple aspects of family well-being.

### 5.2. Practical Implications

From an applied perspective, these findings provide actionable insights for developing family-centered interventions that enhance well-being. Strengthening family function and promoting shared meals can be effective strategies for fostering emotional bonds, improving dietary habits, and enhancing psychosocial health. Policymakers and healthcare providers should design community-based programs that incorporate shared meals as a practical approach to reinforcing family cohesion and supporting long-term health outcomes.

Educational campaigns aimed at improving dietary behaviors should integrate family-centered activities, such as collaborative meal preparation and communal dining, to highlight their protective role in maintaining healthy lifestyles. These initiatives could be particularly beneficial in addressing modern health challenges, including obesity, mental health concerns, and social isolation, by leveraging the benefits of strong family dynamics [46,47]. Grounded in family nursing principles, such programs have the potential to strengthen both individual well-being and overall family resilience.

### 5.3. Limitations and Directions for Future Research

This study provides valuable insights into the relationships among family function, shared cooking and dining practices, psychological flourishing, and family well-being, but certain methodological limitations should be acknowledged. The cross-sectional design limits our ability to determine causal relationships, making it difficult to assess how family interactions evolve over time. Future research should incorporate longitudinal or experimental designs to clarify the temporal and causal dynamics of these relationships.

Reliance on self-reported data may introduce bias related to social desirability or subjective interpretation. Including multi-informant reports or objective behavioral measures in future studies could enhance the validity and reliability of findings. Different family members may experience the same family mealtimes differently, leading to variations in well-being outcomes. Factors such as individual personalities, family roles, and interpersonal dynamics may influence how family interactions impact psychological flourishing and relational satisfaction. Future studies should explore these subjective experiences to better understand how shared activities contribute to well-being at both individual and family levels.

Although the sample provides meaningful insights, its relative homogeneity suggests the need for broader representation. Examining socioeconomic, cultural, and technological factors could provide a more comprehensive understanding of family interactions across diverse populations. Future research should also consider how digital tools, remote communication, or modern meal-sharing technologies influence family cohesion and well-being in evolving social contexts. By addressing these limitations, future studies can enhance methodological rigor and generalizability, further informing family-centered interventions and nursing practices.

## 6. Conclusions

This study examined the relationships among family function, family cooking and dining practices, psychological flourishing, and family well-being, emphasizing the family as a holistic system in family nursing science. The findings highlight the critical role of family function in enhancing psychological flourishing, which serves as both a mediator and moderator in the relationship between family dynamics and well-being. Additionally, shared cooking and dining practices emerged as key interventions for strengthening emotional bonds, improving dietary health, and promoting overall family well-being.

The study reinforces the central role of family interactions in fostering family cohesion, relational harmony, and self-actualization, all of which contribute to holistic health outcomes. Shared meals provide a practical and adaptable approach to strengthening family connections and emotional stability while encouraging balanced nutrition. These insights align with family nursing principles, which emphasize systemic assessments and interventions that support the family as a unit. Recognizing cultural, socioeconomic, and structural variations in family contexts is essential for adapting these interventions to diverse populations.

Future studies could further examine how family interactions and well-being evolve over time, particularly through longitudinal and cross-cultural research.

In conclusion, this study advances our understanding of how family dynamics and shared activities contribute to holistic well-being, offering empirical support for family-centered interventions that promote self-actualization and long-term family health. By framing the family as an interconnected system, these findings provide a strong foundation for developing effective family nursing strategies that enhance sustainable health outcomes and overall family well-being.

## Figures and Tables

**Table 1:** Factor loadings for the Family Function Scale.

Item Description	Factor 1 (Flexibility and Adaptability)	Factor 2 (Intimacy and Harmony)
When problems arise in my family, members reconcile and compromise.	0.863	
When problems arise, my family accepts everyone’s suggestions.	0.798	
In my family, everyone actively participates in major decisions.	0.786	
My family members easily relieve stress and adjust their emotions.	0.773	
My family tries new ways to solve problems.	0.761	
My family members can freely express their opinions.	0.748	
My family effectively uses support systems within the family or from friends.	0.744	
In my family, everyone plays their role well.	0.729	
My family members express their needs in a timely manner.	0.688	
Family members take turns sharing household responsibilities.	0.566	
I think family harmony is the top priority.		0.724
I enjoy doing things with my family, such as dining together.		0.647
I think my family relationships are very close.		0.595
My family members are willing to work hard for the happiness of our family.		0.597
I spend leisure time with my family doing activities.		0.557
I believe my family will always support each other during difficult times.		0.535

**Table 2:** Factor loadings for the Family Cooking and Dining Practices Scale.

Item Description	Factor 1 (Interaction Quality)	Factor 2 (Participation Frequency)
Cooking together strengthens our family bond.	0.874	
Preparing meals allows us to communicate better as a family.	0.822	
Family members share ideas during meal preparation.	0.803	
Dining together brings our family closer emotionally.	0.788	
Meal preparation teaches family members to work together.	0.765	
Our family enjoys discussing daily life during meals.	0.744	
Our family eats together at least three times a week.		0.712
Family members participate in cooking at least twice a week.		0.691
We plan meals together as a family regularly.		0.658
Our family schedules meals to accommodate everyone.		0.634

**Table 3:** Factor loadings for the Psychological Flourishing Scale.

Item Description	Factor Loading
I feel that I am a part of something greater than myself.	0.856
My relationships contribute to my personal growth.	0.835
I feel a sense of purpose in my family interactions.	0.814
I am deeply engaged in activities that are meaningful to me.	0.802
I feel supported by my family in achieving my personal goals.	0.789
My family relationships help me feel emotionally balanced.	0.775
I derive a sense of satisfaction from contributing to my family’s well-being.	0.762
I feel that my family environment fosters personal development.	0.742

**Table 4:** Factor loadings for the Family Well-Being Scale.

Item Description	Factor Loading
I feel happy and satisfied with my family life.	0.841
My family has a harmonious relationship.	0.824
My family environment supports emotional well-being.	0.812
My family members value and respect each other.	0.789
I feel supported by my family in times of need.	0.776
My family communicates openly and effectively.	0.754
My family encourages personal growth and mutual understanding.	0.732
I feel a strong sense of belonging within my family.	0.721
My family maintains a balance between individual needs and collective goals.	0.702
My family works together to overcome challenges.	0.689

**Table 5:** Demographic characteristics of the study participants (*n* = 461).

Demographic Variable	Categories	Sample Size (*n*)
Age	31–40: 27.46%, 41–50: 52.93%	138 (31–40), 323 (41–50)
Gender	Male: 31.88%, Female: 68.12%	147 (Male), 314 (Female)
Family Structure	Nuclear Family: 58.35%, Extended Family: 41.65%	269 (Nuclear), 192 (Extended)
Education Level	University Degree or Higher: 63.57%, Below University: 36.43%	293 (Higher), 168 (Below)
Participation in Cooking/Dining Activities	≥3 Times/Week: 75%, <3 Times/Week: 25%	346 (≥3), 115 (<3)

**Table 6:** Regression results for family function and flourishing.

Variables	β (Standardized Coefficients)	*t*-Value	*p*-Value	R^2^	Adjusted R^2^
Family Function	0.45 *	8.42	<0.001	0.32	0.31
Constant	-	-	-	-	-

Note: * *p* < 0.001 indicates statistical significance.

**Table 7:** Regression results for family cooking practices and well-being.

Variables	β (Standardized Coefficients)	*t*-Value	*p*-Value	R^2^	Adjusted R^2^
Family Cooking and Dining Practices	0.38 *	7.64	<0.001	0.27	0.25
Constant	-	-	-	-	-

Note: * *p* < 0.001 indicates statistical significance.

**Table 8:** The role of psychological flourishing in linking family function to family well-being.

Path	β (Standardized Coefficients)	*t*-Value	*p*-Value	95% CI
Family Function → Family Well-Being	0.34 *	7.12	<0.001	[0.25, 0.43]
Family Function → Psychological Flourishing	0.45 *	8.42	<0.001	[0.36, 0.54]
Psychological Flourishing → Family Well-Being	0.29 *	6.25	<0.001	[0.18, 0.39]
Indirect Effect (via Flourishing)	-	-	-	[0.12, 0.28]

Note: * *p* < 0.001 indicates statistical significance. Bootstrap confidence intervals (CIs) are based on 5000 resamples.

**Table 9:** Psychological flourishing as a moderator between family function and well-being.

Path	β (Standardized Coefficients)	*t*-Value	*p*-Value	95% CI
Family Function → Family Well-Being	0.34 *	7.12	<0.001	[0.25, 0.43]
Psychological Flourishing → Family Well-Being	0.29 *	6.25	<0.001	[0.18, 0.39]
Interaction Term (Family Function × Psychological Flourishing)	0.15 *	3.45	<0.001	[0.08, 0.22]

Note: * *p* < 0.001 indicates statistical significance. Bootstrap confidence intervals (CIs) are based on 5000 resamples.

## Data Availability

Due to research ethics considerations, the data cannot be made available.
